# Synthesis of manganese molybdate/MWCNT nanostructure composite with a simple approach for supercapacitor applications

**DOI:** 10.1039/d2ra04691a

**Published:** 2022-09-29

**Authors:** Kian Yousefipour, Rasoul Sarraf-Mamoory, Shadi Mollayousefi

**Affiliations:** Department of Materials Engineering, Tarbiat Modares University Tehran Iran k.yousefipour@modares.ac.ir rsarrafm@modares.ac.ir

## Abstract

Recently, magnesium molybdate materials have attracted scientific attention for application in supercapacitor devices due to advantages like low synthesis cost and good redox reactions. Nevertheless, these materials endure low electrical conductivity leading to inferior electrochemical performance. To eliminate this drawback, we prepare a composite powder containing magnesium molybdate and functionalized carbon nanotubes (MMO/C) using a simple process to improve the supercapacitive properties. The results proved an electrostatic interaction between the two components of the composite powder, which contains 18–30 nm magnesium molybdate nanoparticles. A crystal model related to magnesium molybdate powder (MMO) was simulated, illustrating that MnO_6_ octahedra are formed next to MoO_4_ tetrahedra. The mesoporous structure of both powders was confirmed whereas the specific surface area of the MMO was enhanced by 69.9% to 36.86 m^2^ g^−1^ in the MMO/C powder with more electroactive sites. The higher electrical conductivity of the MMO/C electrode was proved using electrochemical impedance spectroscopy (EIS) results, with the MMO/C electrode achieving a specific capacitance of 571 F g^−1^ at 1 A g^−1^ current density, improved by more than 4.5 times that of the MMO. Furthermore, the rate performance and cycling stability of the MMO/C electrode reached 87% and 85.2%, respectively. Finally, a two-electrode energy storage device (MMO/C//AC) was assembled. It reveals a specific capacitance of 94.7 F g^−1^, a maximum energy density of 29.6 W h kg^−1^ at a power density of 660.1 W kg^−1^, and cycling performance of 84.3% after 2000 cycles. As a result, the resulting data demonstrate that the MMO/C electroactive material has promising abilities in capacitive energy storage systems.

## Abstract 2

A comparative study on the supercapacitive properties of MnMoO_4_/MWCNT and MnMoO_4_ was conducted to assemble a high-performance supercapacitor device for future energy technologies.

## Graphical abstract

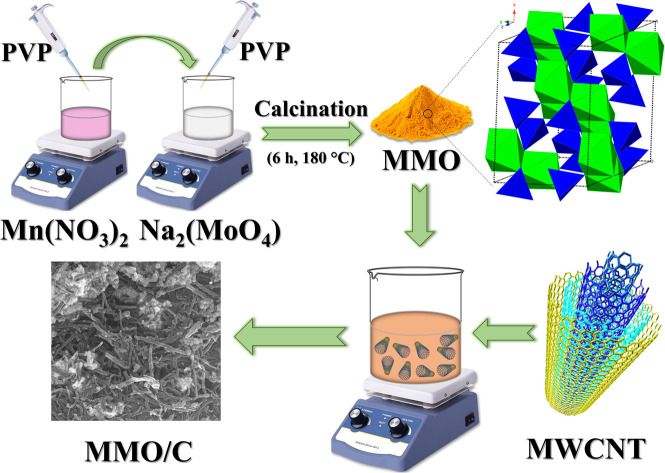

## 1. Introduction

The rising demand for green energy and the worry about the fast diminishing of conventional fossil fuel has caused the research on energy storage to develop toward alternative energy sources.^1^ Furthermore, increasing tendencies to manufacture electronic apparatuses have attracted attention to these types of sources.^2^ In the last decade, electrochemical capacitors (supercapacitors) have been considered a good choice that has attracted significant interest owing to quite a few advantages like more favorable energy and power densities than traditional capacitors and greater power densities than batteries and fuel cells. These devices possess outstanding properties such as high specific capacitance, long cycling stability, good rate performance, fast charge/discharge capability, reliability, and low cost.^3–6^

There are two main charge storage mechanisms in supercapacitors: first, the electrical double layer mechanism related to the separation of charges at the electrode/electrolyte interface, and second, the faradaic redox reaction mechanism ascribed to electron and ion exchange on the noted interface. The former supercapacitors, usually made of carbon-based materials, are denoted as electrical double layer capacitors (EDLCs), and the latter, made of different transition metal oxides or conducting polymers, are known as pseudocapacitors.^2,7^ Even though pseudocapacitors prepare higher capacity and energy density, caused by faradaic fast redox reactions, these materials still suffer from low electrical conductivity. Hence, to employ the best benefits of the supercapacitive performances, applying a helpful technique for forming an efficient material is essential.

The supercapacitor electrode voltage can change depending on the electrolyte type. Accordingly, two common electrolyte types utilized in supercapacitors are organic and aqueous solutions. The organic electrolytes suffer from low ionic conductivity, inflammable nature, environmental incompatibility, overcharging during the process, and high prices. In contrast, an aqueous electrolyte yields more benefits than the organic one, like a more compatible nature, lower cost, higher ionic conductivity, and reduced heat generation.^8^ According to this, employing aqueous electrolytes provide better energy storage performances. On the other hand, the breakdown voltage of water in these electrolytes is around 1.2 V, and the potential window is restricted to lower values.^9^

According to the literature,^10^ binary metal oxide materials have been considered an encouraging alternative due to their wide voltage windows, enhanced cyclability, remarkable electrical conductivity, and more electroactive sites. Additionally, among these oxides, magnesium molybdates can be assessed as a beneficial active material to raise electrochemical properties due to oxidation states coming from Mn ions and conductivity from Mo.^11^ This material suffers from two main drawbacks, low electrical conductivity and surface destruction during redox reactions. Employing carbon-based materials seems crucial to deal with the limitations.^12,13^

Carbonaceous materials possess high mechanical flexibility, large specific surface area, good aspect ratio, and excellent electrical conductivity.^14^ Recent studies indicate that among these materials, multiwalled carbon nanotubes (MWCNTs), with their superior properties, are utilized to employ in EDLCs supercapacitors.^15^ Thus, using carbon nanotubes beside binary oxide materials, having high electrochemical properties and low cast, has encouraged the research community. For example, Ghosh *et al.*^16^ reported hydrothermally prepared MnMoO_4_/graphene composite electrode with 84% cycle life and 364 F g^−1^ capacitance at 2 A g^−1^ current density, whereas the produced pure MnMoO_4_ outputted 234 F g^−1^ at the same condition. This research states that the electrical conductivity of pure binary oxide and composite samples delivers 4.27 × 10^−3^ S cm^−1^ and 19.43 S cm^−1^ conductivities, respectively, justifying higher capacitance of the composite electrode. In another study investigated by Thangappan *et al.*^17^ the specific capacitance of MnMoO_4_/graphene nanocomposite improved by 49.7% to 302.08 F g^−1^ at a current density of 0.1 A g^−1^. Referring to this study, the chemical interaction of Mn^2+^ and MoO_4_^2−^ led to the formation of MnMoO_4_ crystalline nanoparticles, and at the same time, GO sheets are reduced using citric acid as the reducing agent attached to the binary oxide component. Thus, the presence of a large number of defects along with higher conductivity created by graphene prepared more active regions to enhance energy storage. Based on^18^ research, an electrospinning-carbonization-thermal annealing method was implemented to synthesize MnMoO_4_/carbon nanofibers composite. The specific capacitance of the electrode was obtained 389.7 F g^−1^ at 0.1 A g^−1^, and the cycle life results showed capacitance retention of 92.1% after 5000 cycles. Mu *et al.*^19^ reported hydrothermally treated hierarchical CNT/rGO on MnMoO_4_ nanosheets. The prepared device with 87.5% cycling stability demonstrated an energy and power density of 59.4 W h kg^−1^ and 1367.9 W kg^−1^, respectively.

Co-precipitation is one of the simple and cost-effective approaches mainly employed caused by producing a uniform and high purity ceramic oxides along with ease of adjusting experimental parameters.^20,21^ On the other hand, the mechanical mixing method has some benefits like simplicity, flexibility, and unit operation.^22^ As a result, introducing a MnMoO_4_/MWCNT electrode material with a simple technique could proficiently enhance the energy storage properties of the MnMoO_4_ with a simple synthesizing process enhancing its most common drawbacks.

In this study, we have prepared simple co-precipitation and mixing approaches to achieve magnesium molybdate (MMO) and magnesium molybdate/multiwalled carbon nanotube (MMO/C) electroactive powders for being used as electrode materials to investigate their supercapacitive performance. Our studies clearly showed that by applying the MMO/C powder, the capacity of the MMO boosted from 125 to 571 at 1 A g^−1^ current density and by 582 at 10 mV s^−1^ scan rate. Furthermore, the cycle life and rate performance of the composite powder reached 85.2% and 87%, by 44.9% and 28% higher than the ones of the MMO powder, respectively.

## 2. Experimental

### 2.1. Materials

The as-obtained MWCNT (purity > 95%) was received from Cheap Tubes Inc. (USA). Mn(NO_3_)_2_·4H_2_O (purity ≥ 97%), Na_2_(MoO_4_)·2H_2_O (purity ≥ 99.5%), and poly(vinyl pyrrolidone) (PVP) were purchased from Sigma-Aldrich. Nickel foam was prepared from LSS Company (Singapore). Poly(vinylidene fluoride) (PVDF), carbon black (CB), activated carbon (AC), and dimethylformamide (DMF) were procured from Sigma-Aldrich. The water utilized in all experimental sections was deionized (DI) with a conductivity of 0.1 μs cm^−1^.

### 2.2. Methods

The MMO and MMO/C powders synthesis were conducted as follows: first, 2.25 g Na_2_(MoO_4_)·2H_2_O and 2.32 g Mn(NO_3_)_2_·4H_2_O were separately prepared in 100 mL DI-water and constantly mixed for about 30 min. At the same time, 0.02 g PVP was dissolved in 20 mL DI-water and added equally to the above solutions, 1 mL for each. Subsequently, the magnesium nitrate solution was dropwise added to the sodium molybdate under continuous stirring for up to 30 min. The obtained milky solution was separated and then rinsed well with ethanol and DI-water mixture several times *via* centrifugation under 1000 rpm for 15 min. Finally, the as-synthesized material was perfectly dried at 60 °C for 10 hours, named hydrated MnMoO_4_. To achieve the final product (MMO), the prepared precursor was calcined at 900 °C for 2 h.

Second, the purchased MWCNTs were calcined at 380 °C for 2 h to eliminate the amorphous carbon materials. Thereafter, this powders were dispersed entirely in 7 M HNO_3_ under ultrasonic conditions within 15 min and refluxed at 120 °C for 10 h. The mentioned powders were eventually washed and then dried under heating. For producing the composite powder (MMO/C), 120 mg of the MMO powder, synthesized in the first part, was mixed in 50 mL DI-water for 20 min. The noted solution was dropwise poured into 50 mL functionalized MWCNTs solution with a concentration of 2 mg mL^−1^ under stirring, and then the mixing was continued within 72 h at ambient temperature. The washing and drying processes were carried out the same way as in the first part.

### 2.3. Characterization

The crystallographic phase structure of the as-prepared materials was performed by X-ray diffraction (XRD) technique with Co-Kα radiation. The thermal characteristic of the MMO powder was appointed using an STA 503 BÄHR device at the range of 40–900 °C and a heating rate of 10 °C min^−1^ in the ambient atmosphere. In order to observe the surface morphology of both powders, a field emission scanning electron microscope (FESEM) with a MIRA3 TESCAN model was employed. The specific surface area of the materials was measured by utilizing the adsorption–desorption isotherms of nitrogen *via* the Brunauer–Emmett–Teller (BET), and pore size distribution was computed by the Barrett–Joyner–Halenda (BJH) method (Micrometrics TriStar II 3020). In addition, fourier transform infrared (FTIR) spectroscopy (by PerkinElmer–Frontier) was utilized to recognize the functional groups on the surface of materials.

### 2.4. Cell preparation and electrochemical measurements

The supercapacitive properties of the powders were investigated in both three- and two-electrode cell setups. The nickel foam was first washed with acetone for 15 min, and later a 2 M HCl solution under sonication was handled for 30 min to exclude debris and surface oxides. Finally, it was washed with acetone and ethanol. For assembling the three-electrode system, the as-prepared electroactive materials were mixed with PVDF and CB powders in DMF solution, whereas their mass ratio was 80 : 10 : 10, respectively. After mixing, the acquired homogeneous paste was pressed onto the nickel foam with a dimension of 1 × 1 cm^2^, denoted as working electrodes. The electrodes containing platinum and calomel were considered the counter and reference, respectively, in the three-electrode setup. The working electrode was prepared to produce the asymmetric two-electrode device, similar to what was mentioned, placed as the positive pole and AC as the negative. A piece of cellulose paper was positioned between two poles of the cell in order to separate them. The electrochemical features of the working electrodes were measured in a 2 M KOH aqueous solution in both systems. An OrigaFlex potentiostat workstation was implemented to measure the electrochemical characterizations using electrochemical impedance spectroscopy (EIS) (with a frequency range of 10 mHz to 100 kHz), cyclic voltammetry (CV), and galvanostatic charge–discharge (GCD) methods.

## 3. Results and discussion

**Fig. 1:**
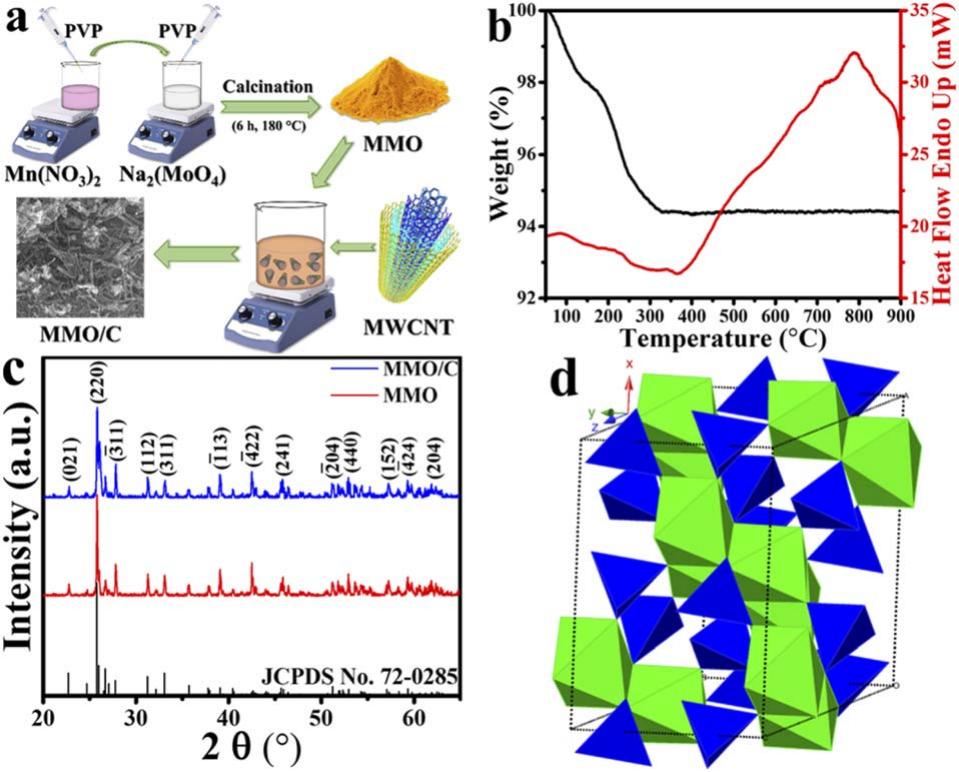
(a) Schematic of synthesis procedure of the MMO and MMO/C powders, (b) TGA-DSC curves of the MMO sample, (c) XRD patterns of the MMO and MMO/C powders along with reference pattern of MnMoO_4_, and (d) crystal structure of the MMO powder.

The simple schematic illustration of both MMO and MMO/C synthesis procedure is depicted in Fig. 1a. Thermogravimetric analysis (TGA) of the as-synthesized hydrated MnMoO_4_ nanopowder was measured from 40 to 900 °C to obtain the optimal calcination temperature of the nanoparticles and the possible phase transformations during the heating. From Fig. 1b, the TG curve, it is observable that two significant stages are there up to 400 °C, from 100 to 200 °C and 300 to 400 °C, proved with two broad endothermic peaks in this temperature range in the differential scanning calorimetry (DSC) curve. The overall weight loss, around 5.6% by 400 °C, can be related to the removal of structural and absorbed water molecules, debris, and volatile surface materials. Moreover, it is recognizable from the DSC curve that there is a characteristic intense exothermic peak at around 780 °C, corresponding to the phase transformation of hydrated MnMoO_4_ solid solution into MnMoO_4_ compound, similar to what was discussed elsewhere.^23,24^ Accordingly, the calcination temperature for producing MnMoO_4_ nanostructure was chosen at 900 °C in this study, as depicted in the experimental section.

In order to confirm the successful synthesis and assess the phase composition of the prepared powders, the XRD analysis was utilized, as shown in Fig. 1c. Considering the data displayed, it is revealed that the powder patterns are well-matched with JCPDS card no. 72-0285, which ascribes to monoclinic manganese molybdenum oxide. By carefully comparing the patterns in Fig. 1c, it is clear that there are no extra XRD peaks in the patterns of the MMO and MMO/C samples than the pure material, indicating the high purity of the nanoparticles.

Compared to what was presented in the MMO pattern, the XRD pattern of the composite sample revealed a broader peak at around 2*θ* ≈ 26. This peak corresponds to the (002) plane of MWCNTs and discloses the hexagonal graphite structure.^25^ Hence, the construction of the composite by coupling MnMoO_4_ nanoparticles with MWCNTs was evidently proved. Meanwhile, a comparative evaluation with the pure standard pattern represents that some XRD peaks of both MMO and MMO/C powders are more intense than usual, ascribed to those crystallographic plans with more X-ray exposure.^26^ The average grain size measurements obtained by the Scherrer equation from the XRD patterns were calculated to be 18.7 and 16.9 nm for the MMO and MMO/C powders, respectively. As one can see, although the mean crystallite size in the MMO/C is lower, there is no significant grain size difference between the powders.

Based on the XRD results, the MMO crystal delivers a monoclinic structure and *C*2/*m* space group containing Mn and Mo atoms coordinated as MnO_6_ octahedra and MoO_4_ tetrahedra, respectively. Fig. 1d, achieved using the CrystalMaker software, indicates the polyhedral model related to the noted structure extracted from the literature.^27^ According to what was formerly concluded from the TG-DSC results, it is clear from the XRD patterns that the obtained calcination temperature, around 900 °C, is favorable for attaining the high purity MnMoO_4_ powder.

**Fig. 2:**
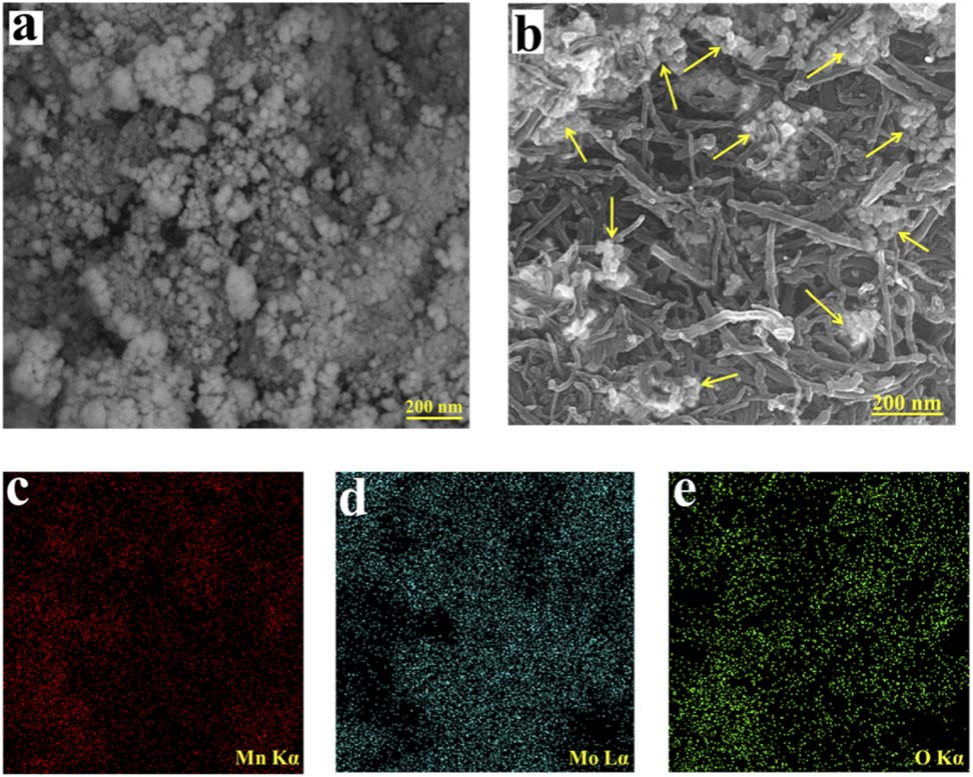
FESEM images of the: (a) MMO and (b) MMO/C followed by elemental mapping images of (c) Mn, (d) Mo, and (e) O.

For evaluating the morphologies of the powders, the FESEM technique was used. Fig. 2a and b represent the FESEM images of the MMO and MMO/C electroactive materials, respectively. The morphology of the MMO powder consists of a large sum of tiny particles in conjunction with each other to produce a uniform nanostructure. The particles composed of agglomerates form a homogeneous structure with a cluster-like morphology. Measured from FESEM images, the mean size of the manganese molybdate particles was calculated to be approximately in the range of 20 to 210 and 18 to 30 nm in the MMO and MMO/C particles as indicated in Fig. 2a and b. This occurrence demonstrates that, caused by the mechanical mixing procedure for 72 h and the presence of MWCNTs between the particles, some agglomerates were broken, and the average particle size decreased. Thereby, the noted measurements increase the number of active sites on the electrode surface, improving the supercapacitive properties.

However, the FESEM image of the MMO/C powder (Fig. 2b) shows that MWCNT nanomaterials with an approximate average diameter of 20–30 nm and length of 200–450 nm locate between oxide nanoparticles. The improved electrical conductivity of the composite comes from MWCNTs,^28^ leading to the path of ion penetration among nanoparticle voids facilitates, and also energy storage increases. In this way, the favorable benefits of each component collected to form the composite powder cause the supercapacitive performance to increase compared to the other powder, which will be discussed in the electrochemical part. Fig. 2c–e indicate the elemental mapping images related to Mn, Mo, and O, correctly showing the uniform distribution of these elements in the MMO powder.

**Fig. 3:**
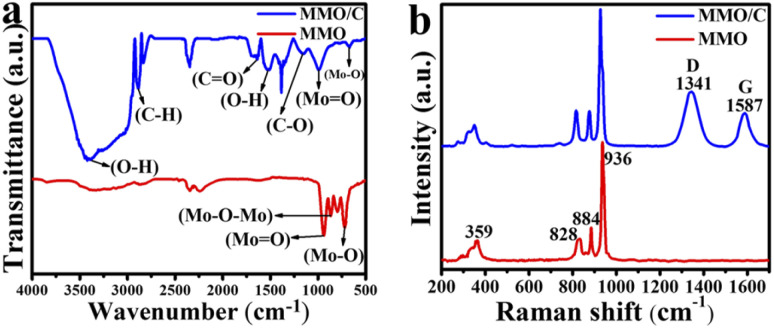
(a) FTIR and (b) Raman spectra of both active materials.

The FTIR spectra were used for analyzing surface functional groups. Fig. 3a shows the FTIR results of the MMO and MMO/C powders. The synthesized MMO sample presents three main peaks like other molybdate-based studies.^29^ The strong bound at about 940 cm^−1^ is related to the stretching vibration mode of the Mo=O functional group. Also, the peak at around 867 cm^−1^ corresponds to the characteristic bending mode of Mo–O–Mo. Finally, the strong bond at 718 cm^−1^ is attributed to the stretching vibration mode of Mo–O placed on the MoO_4_ tetrahedral structure discussed and modeled previously. For the composite sample, the number of characteristic peaks correlated to the carbonaceous material shown in Fig. 3a is five. The intense characteristic peaks at 1633 and 1155 cm^−1^ are ascribed to stretching vibration of C=O and C–O bonds, and these functional groups are related to carboxylic acid (–COOH) groups^30,31^ due to the oxidation of a number of carbon atoms on the surface of MWCNTs. The peaks at 1523 and 3414 cm^−1^ are assigned to O–H in the –COOH groups. A strong bond at about 2886 cm^−1^ is related to the C–H stretching vibration of carbonyl groups.^32^

Raman spectroscopy was collected to further study the structure of both powders. Fig. 3b displays the Raman spectra of the MMO and MMO/C. The spectrum of the MMO discloses a sharp bond at 936 cm^−1^ and three bonds with less intensity at 884, 828, and 359 cm^−1^. These peaks are three primary characteristic bands of the MMO.^33^

In the CNT-based composite, along with the red-shift compared to the MMO peaks, two extra peaks clearly appear. By carefully tracking the positions of the main peak of the MMO at 936 cm^−1^, it is clear that the value reaches 927 cm^−1^ in the composite one. Moreover, a similar behavior occurs for the other three peaks. The mentioned red-shift can be ascribed to an electrostatic interaction between magnesium molybdate and functional groups placed on the surface of MWCNTs during the preparation process. Owing to the positive effects of each composite component, the noted occurrence can increase the overall electrochemical properties of the composite powder.^34^ Besides, two additional typical bands are in the MMO/C sample, the D band at 1341 cm^−1^ and the G band at 1587 cm^−1^. It is known that the D band is related to crystal defects and disordered structure in carbon lattice, and the G band is attributed to the carbon atoms with sp^2^ hybridization.^35^ The *I*_D_/*I*_G_ ratio reveals the degree of structural disorder in the composite powder. This ratio was calculated at about 1.14, suggesting defects of carbon structure in the MMO/C. The obtained value greater than one results in higher active sites for energy storage and more interactions in the electrode/electrolyte interface.^36^

Finally, it is interesting to notice that the FTIR and Raman results strongly confirm the synthesis of both the composite and oxide powders.

**Fig. 4:**
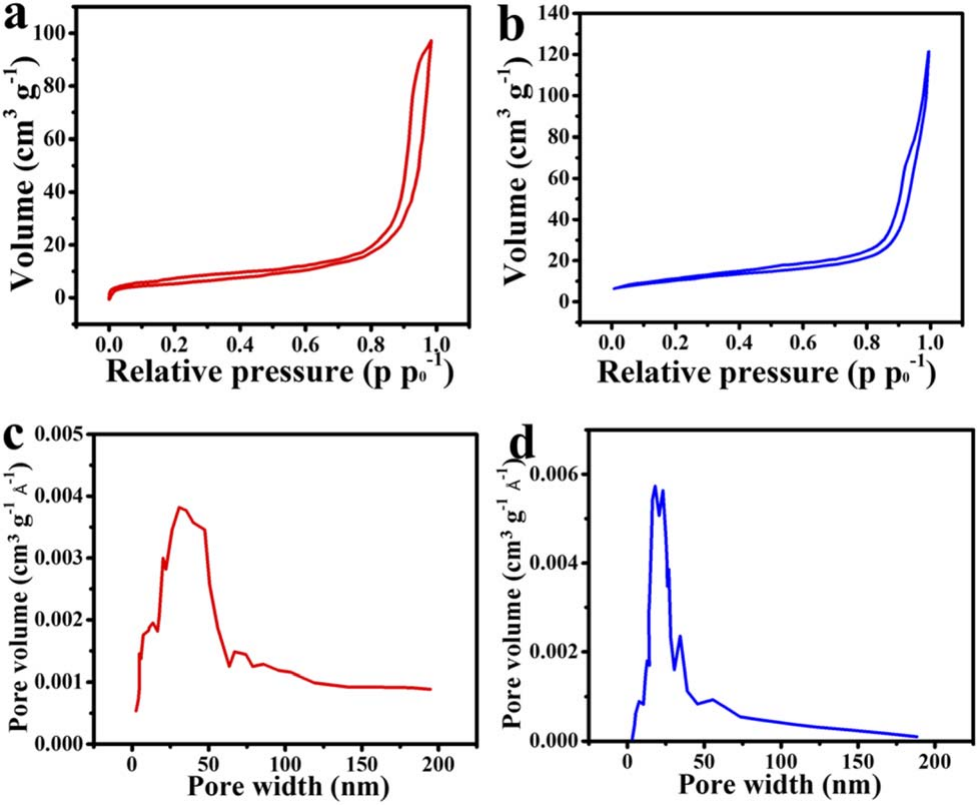
Curves of: adsorption/desorption isotherm of the (a) MMO and (b) MMO/C, and pore size distribution of the (c) MMO and (d) MMO/C powders.

The BET and BJH techniques were implemented to analyze the specific surface area and pore size distribution of the MMO and MMO/C powders. The N_2_ adsorption–desorption isotherms of the powders are illustrated in Fig. 4a and b. According to the hysteresis loop shape of both powders, the isotherms can be attributed to the type IV IUPAC isotherm in which there are quite a few mesopores within the materials. The BET results indicate that the specific surface areas related to the MMO and MMO/C powders were found to be around 21.7 and 36.86 m^2^ g^−1^, and the pore volumes around 0.149 and 0.179 cm^3^ g^−1^, respectively. The more the specific surface area, the higher the specific capacitance will be, arising from the high surface area value of MWCNTs directly affecting it.

Furthermore, the BJH curves of the powders (Fig. 4c and d) disclosed the pore size distributions in the MMO and MMO/C materials obtain 35 and 19 nm, respectively, further implying the average pore sizes are in the mesoporous range (2–50 nm). Besides, more mesopores can further improve the power density, thus related to faster movements of electrolyte ions into the surface of the electrode, as already discussed.^37^ Accordingly, the penetration of electrolyte ions into the MMO/C mesopores, with smaller pore size, causes the quality of redox reactions, the charge transfer, and also supercapacitive performance to improve.

**Fig. 5:**
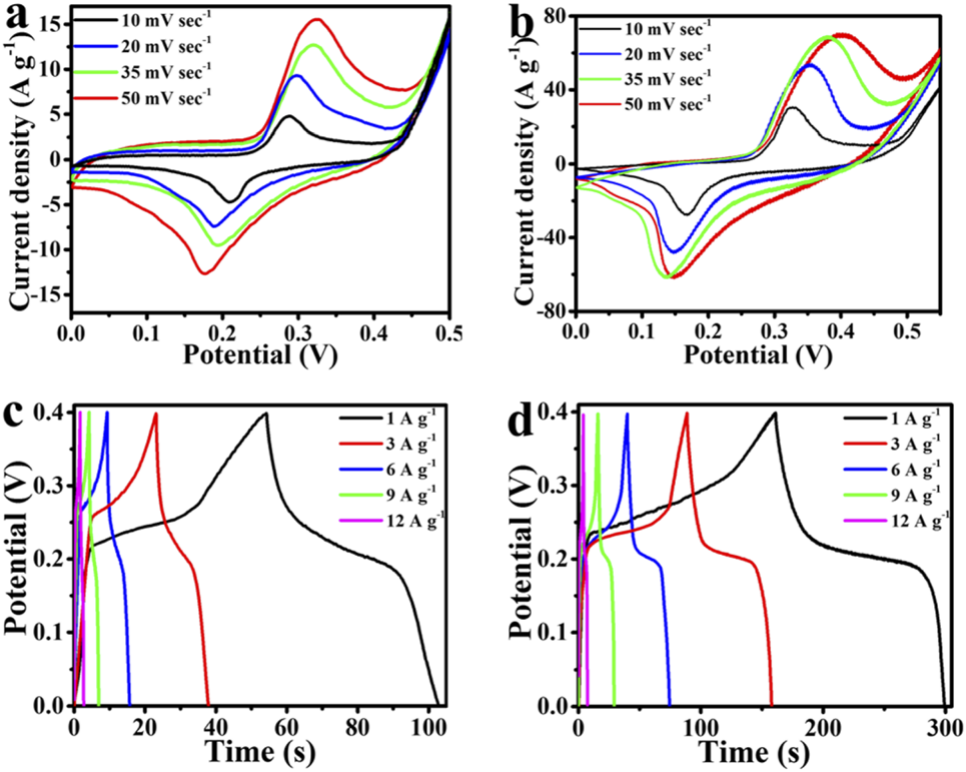
Supercapacitive properties of the electrodes: CV curves of the (a) MMO and (b) MMO/C, GCD curves of the (c) MMO and (d) MMO/C.

**Fig. 6:**
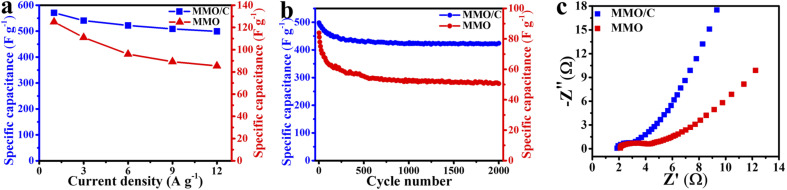
Comparison of (a) rate performance, (b) cycling stability under 2000 cycles, and (c) EIS data of the MMO and MMO/C electrodes.

The supercapacitive properties of the MMO and MMO/C powders are represented in Fig. 5 and 6. Fig. 5a and b indicate the CV curves assigned to both electrodes at 10, 20, 35, and 50 mV s^−1^ scan rates, respectively. It is evident that the potential windows of the electrodes are normally determined by increasing the potential until there is the occurrence of hydrogen evolution reaction (HER) on the negative side and oxygen evolution reaction (OER) on the positive side of the curve.^38^ The HER and OER denote the breakdown of aqueous electrolytes, which will be shown by a sharp increase in the current density. Thus, the best potential windows in the CV curves of the MMO and MMO/C samples were determined from 0 to 0.5 V and 0 to 0.55 V, respectively, as illustrated in Fig. 5a and b.

A pair of redox peaks is observed by carefully assessing all CV curves, implying redox reactions happen during the cycles.^39^ It is suggested that^16,40^ an electron transfer between Mn^2+^ and Mn^3+^ ions in the electrochemical double layer is responsible for redox activities leading to pseudocapacitance behavior.^11^ On the basis of Fig. 5a and b, ascribed to oxidation of Mn^2+^ to Mn^3+^, the peaks at about 325 mV in the MMO powder and at about 402 mV in the MMO/C powder are formed, whereas the scan rate value is 50 mV s^−1^. In the same scan rate, the reduction process arising from Mn^3+^ to Mn^2+^ is conducted at about 175 and 145 mV in the MMO and MMO/C samples, respectively. In this case, Mo role is to enhance the electrical conductivity of the active materials and cannot take part in the ion exchange phenomenon and eventually enhance the specific capacitance.^41^ Meanwhile, by increasing the scan rates in the CV curves of electrodes, the peaks at the oxidation and reduction branches of the curves shift toward the right and left, respectively. This case can be related to the more significant electron and ion transfer resistance at higher scan rates under the diffusion-controlled process.^42,43^

The GCD curves related to both MMO and MMO/C electroactive materials at 1, 3, 6, 9, and 12 A g^−1^ current densities show the maximum discharge time takes place at 1 A g^−1^ current density, as shown in Fig. 5c and d. Herein, the maximum capacitance values of the MMO and MMO/C electrodes, calculated from the discharge section of the GCD curves according to what presented elsewhere,^26^ were obtained at 125 and 571 F g^−1^, respectively. With more calculations in the MMO/C electrode, the values at 3, 6, 9, and 12 A g^−1^ current densities achieve 541, 522, 509, and 499 F g^−1^ capacitance, respectively. Although the typical type of the curves does not vary notably, the area under any curves increases with declining the current density, occurring by increasing the specific capacitance. The IR-drop data of the MMO and MMO/C powders at 1, 3, 6, 9, and 12 A g^−1^ current densities were obtained from the GCD curves. The IR-drop is because of the electrodes’ internal resistance, the ionic conductivity of the electrolyte, and ionic movement of the electrodes.^41^ Our evaluations show the acquired IR-drops rise as the current densities do as well. The MMO and MMO/C IR-drops are in the range of 9.4 to 49.6 and 2.1 to 31.9 mV, respectively, demonstrating better conductivity of the MMO/C electrode during electrochemical assessments.

A comparative assessment of the specific capacitances obtained from CV and GCD curves for the MMO/C electrode is exhibited in Table 1. As one can see, the specific capacitance related to the composite powder is as high as 571 and 582 F g^−1^ at 1 A g^−1^ current density and 10 mV s^−1^ scan rate, respectively, indicating well-matched specific capacitances. Also the values were compared to the other literature studies. It is noteworthy that the capacitance can be affected by the initial materials, synthesis procedure, and the conditions of electrochemical measurements.

**Table 1:** Comparison of specific capacitance of different MnMoO_4_-based materials with our work

Electrode	Synthesis route	Electrolyte	Current density (or) scan rate	Specific capacitance	Ref.
MnMoO_4_/graphene	Hydrothermal	1 M Na_2_SO_4_	2 A g^−1^	364 F g^−1^	16
CNT/rGO/MnMoO_4_	Hydrothermal	2 M NaOH	2 mV s^−1^	2374.9 F g^−1^	19
MnMoO_4_/PANI	Oxidative polymerization	1 M Na_2_SO_4_	5 mV s^−1^	396 F g^−1^	44
MnMoO_4_/PPy	Oxidative polymerization	2 M KCl	1 A g^−1^, 5 mV s^−1^	313, 462.9 F g^−1^	45
PPy@MnMoO_4_/CFs	Deposition	0.6 M H_2_SO_4_	1 A g^−1^	302 F g^−1^	46
NiCo_2_O_4_@MnMoO_4_	Hydrothermal	2 M KOH	1 mA cm^− 2^	1205.75 C g^− 1^	47
MnMoO_4_	Electrospinning-carbonization-thermal annealing	2 M KOH	1 A g^−1^	156 F g^−1^	18
MnMoO_4_	Hydrothermal	1 M NaOH	5 mV s^−1^	1271 F g^−1^	48
MnMoO_4_	Simple method	NaOH	1 A g^−1^	562 F g^−1^	49
MnMoO_4_	Solid-state chemistry	2 M NaOH	1 A g^−1^	210.2 F g^−1^	24
MnMoO_4_	Hydrothermal	1 M KOH	5 mV s^−1^	376 F g^−1^	50
MMO/C	Electrostatic co-precipitation	2 M KOH	1 A g^−1^, 10 mV s^−1^	571, 582 F g^−1^	Present study

Fig. 6a displays this incident where rate performance curves can be conveyed from the GCD curves of both electrodes. This phenomenon arises because there is less time for ion penetration on the active material surface at higher values of the current densities. At this moment, by decreasing the rate of ion transfer, the redox reactions on the electrode/electrolyte interface cannot be adequately carried out as well as those at the lower current densities.^34^ Therefore, the lower the current densities were applied, the better the redox reactions were done throughout the surface.

As it is apparent in the MMO/C sample, even if the current density reaches 12 A g^−1^, more than 87% of the highest capacity at 1 A g^−1^ remains. In this condition, the corresponding value for the MMO sample is about 68%, by 22% lower. Hence, the rate performance of the MMO/C electrode is higher than the one of the MMO electrode. Evaluating these variations, the higher specific capacitance, by 357%, and rate performance, by 28%, of the composite sample are ascribed to its higher electrical conductivity and ion movement, resulting in a faster kinetic process due to the presence of conductive MWCNT networks.^51^ The specific capacitance of the electroactive materials can also be calculated using the CV data. The computed capacities of the MMO/C electrode at 10, 20, 35, and 50 mV s^−1^ scan rates are 582, 562, 488, and 376 F g^−1^, respectively. Nevertheless, the MMO electrode manifests the capacities of 119, 117, 98, and 93 F g^−1^ at the same scan rates. A complete evaluation of the CV curves of both electrodes indicates that as the scan rates decrease, so inversely do the specific capacitances, suggesting the mobility of the electrolyte ions increases while applying higher scan rates, causing the ionic exchange to decline.^52^ It is specified from the capacitances calculated from both GCD and CV curves that the MMO/C electrode reveals higher specific capacitance than that for the MMO.

Fig. 6b depicts the cycle life curves of the MMO and MMO/C electrodes at the current density of 12 A g^−1^. Altogether, the comparison of the overall capacitance retention of the MMO and composite electrodes indicates the former possesses 58.8% and the latter 85.2% of their initial capacitance values after 2000 cycles. Both electrodes reveal that almost over the first 180 cycles, a more noteworthy decrease in the specific capacitance takes place, according to the report.^16^ When cycle numbers increase from 180 to 700, the correlated capacitances decrease slower than the previous step, and up to 2000 cycles, it stays nearly constant. It seems that the degradation of the active materials due to internal stresses, along with their fast contraction and expansion during cycling, are the main reasons for these changes.^48,53–55^ As a consequence, the higher value of the cycle life obtained in the MMO/C electrode than that of the MMO arises from two main reasons: first, the higher electrical conductivity of MWCNTs applied throughout the electroactive material, which will prove further using the EIS results, and second, lower surface destruction coming from better stress distribution on the overall surface of the active material during cycling.

The EIS technique was used to evaluate further the electrochemical properties of both electroactive materials. Fig. 6c represents the EIS results of the MMO and MMO/C electrodes, shown as the Nyquist plot. The exhibited curves have two principal regions: the high-frequency regions with a semicircle shape and the low-frequency regions with a relatively straight line. The former region (*R*_ct_) is ascribed to charge exchange resistance throughout the electrode/electrolyte interface, and the latter, named Warburg impedance (*Z*_w_), is related to the kinetics of hydroxide ions on the active material surface. Additionally, the value of *x*-intercept (*R*_s_) is attributed to the internal resistance of the powders as the electroactive materials.^56–60^

It is apparent in the EIS curves that the *R*_s_ values of the electrode containing the MMO and MMO/C powders are 2.1 and 1.8 Ω, respectively, demonstrating that the MMO/C reveals higher conductivity and improved mobility of ions.^61^ Meanwhile, the radius of the semicircle region related to the MMO/C electrode is smaller, and as a result, its *R*_ct_ value is lower than that of the MMO, which manifests better ionic exchange on the electrode/electrolyte interface. Besides, the lower Warburg impedance of the MMO/C electrode, at the lower frequency part, shows more excellent hydroxide kinetics for diffusion on the surface of the active material.^47,62,63^ Altogether, the EIS results imply that the MMO/C material contains a good electrostatic connection of the MMO particles and MWCNTs and shows higher ion exchange, electron transfer, and ionic diffusion kinetics at the electrode/electrolyte interface than those of the MMO due to the presence of MWCNTs.

**Fig. 7:**
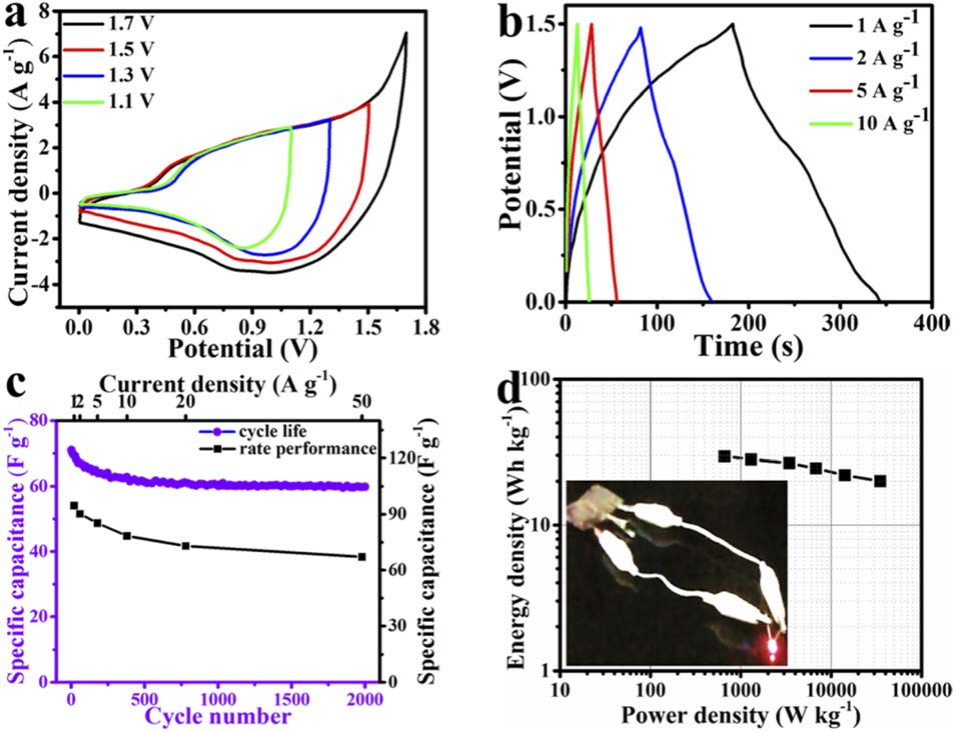
Supercapacitive properties of the MMO/C//AC asymmetric device: (a) CV curves at 50 mV s^−1^ scan rate and various voltage windows, (b) GCD plots at different current densities, (c) cycle life and rate performance, and (d) Ragone plot and lighted up LED by two devices in series.

The MMO/C and AC were applied as the positive and negative electrodes, respectively, while assembled in a two-electrode system (MMO/C//AC). To find the best operating voltage of the device, the CV measurements were conducted at various voltage windows and 50 mV s^−1^ scan rate. Our investigations revealed that the most favorable voltage window is in the 0–1.5 V range, as observed in Fig. 7a. It is stated elsewhere^64^ that a greater operating voltage, in our case 1.7 V, brings about a higher current density due to water splitting and electrolyte decomposition throughout the electrode surface.

Fig. 7b specifies the GCD data at various current densities extracted from the MMO/C//AC asymmetric device. The maximum specific capacitance of the device obtained 94.7 F g^−1^ at 1 A g^−1^ current density *via* these curves. As one can recognize, similar to what was stated in the three-electrode system, the rate performance curve in the device was obtained using GCD plots, as shown in Fig. 7c. Consequently, with increasing current densities, the capacities increase because of the higher penetration resistance resulting in heat loss at higher current densities. Additionally, with its 70.8% capacity retention at 50 A g^−1^, the device discloses a great rate performance, demonstrating rapid electron transfer and good ion penetration coming from its good electrical conductivity.^65^

Fig. 7c also showcases the cycle life curve at a current density of 20 A g^−1^ within 2000 cycles. It is recognizable that the cyclical behavior of two- and three-electrode systems are similar. According to the cyclic life results of the device, about 84.3% of its initial specific capacitance remains after 2000 cycles, which reveals its remarkable stability.

Fig. 7d depicts the Ragone plot of the device at diverse current densities along with an inset image representing the two prepared devices in series lighted up by an LED. The resulting energy and power density data presented in this figure were calculated according to the earlier study.^41^ The energy density values were calculated to be around 29.6, 28.2, 26.6, 24.5, 22.1, and 20 W h kg^−1^ at the power density values of 660.1, 1298.1, 3447.8, 6784.6, 14 184.6, and 34 951.8 W kg^−1^, respectively. On the basis of obtained results, a maximum energy density of 29.6 W h kg^−1^ at a power density of 660.1 W kg^−1^ was computed.

Finally, we studied the supercapacitive and characteristic properties of the MMO and MMO/C electrode materials under a comparative study. The data obtained from the asymmetric two-electrode MMO/C//AC device demonstrate excellent electrochemical abilities to implement in high-performance supercapacitor applications.

## 4. Conclusion

In summary, a simple co-precipitation method was implemented for the synthesis of the MMO powder, and then this material was electrostatically interacted with functionalized multiwalled carbon nanotubes to prepare the MMO/C composite. Both powders were further characterized, and their supercapacitive performances were compared in a three-electrode system. The MMO/C electroactive material maintains a high capacity retention of 85.2% after 2000 cycles and retains the rate performance of 87%. By preparing the composite electrode, the specific capacitance of the MMO was successfully enhanced from 125 to 571 F g^−1^ at 1 A g^−1^ current density and from 119 to 582 F g^−1^ at 10 mV s^−1^ scan rate under identical measurement conditions. Thanks to its electrochemical performance, the MMO/C active material was selected to be evaluated in an assembled device. The outstanding supercapacitance properties of the MMO/C material are related to its higher electrical conductivity, more electroactive sites, and better ion exchange. The resulting data implied that owing to the easy experimental procedure and promising supercapacitive abilities, such a composite powder could be considered a good choice for electronic devices.

## Conflicts of interest

There are no conflicts to declare.

## Supplementary Material
