# Correction: Synthesis of manganese molybdate/MWCNT nanostructure composite with a simple approach for supercapacitor applications

**DOI:** 10.1039/d2ra90128e

**Published:** 2022-12-20

**Authors:** Kian Yousefipour, Rasoul Sarraf-Mamoory, Shadi Mollayousefi

**Affiliations:** Department of Materials Engineering, Tarbiat Modares University Tehran Iran k.yousefipour@modares.ac.ir rsarrafm@modares.ac.ir

## Abstract

Correction for ‘Synthesis of manganese molybdate/MWCNT nanostructure composite with a simple approach for supercapacitor applications’ by Kian Yousefipour *et al.*, *RSC Adv.*, 2022, **12**, 27868–27876, https://doi.org/10.1039/D2RA04691A.

In the original article, the authors regret that compound names were inserted incorrectly. “Magnesium” was used in error, when “Manganese” should have been used. There are 9 instances in the original manuscript where the incorrect compound name was included. Every instance should be replaced with “Manganese”. The authors regret this error. The scientific conclusions of this article remain unchanged.

The Royal Society of Chemistry apologises for these errors and any consequent inconvenience to authors and readers.

## Supplementary Material

